# Routine contrast-enhanced CT screening identifies asymptomatic pulmonary embolism after minimally invasive esophagectomy: incidence and associated factors

**DOI:** 10.1007/s10388-026-01224-0

**Published:** 2026-06-30

**Authors:** Akira Saito, Tomotake Ariyoshi, Satoru Goto, Takeshi Yamashita, Kentaro Motegi, Masahiro Kohmoto, Koji Otsuka, Masahiko Murakami, Takeshi Aoki

**Affiliations:** 1 Division of General and Gastroenterological Surgery, Department of Surgery, Showa Medical University School of Medicine, 1-5-8, Hatanodai, Shinagawa, Tokyo, 142-8666 Japan; 2 Esophageal Cancer Center, Showa Medical University Hospital, Tokyo, Japan; 3 Digestive Diseases Center, Showa Medical University Koto Toyosu Hospital, Tokyo, Japan

**Keywords:** Pulmonary embolism, Venous thromboembolism, Minimally invasive esophagectomy, Esophageal cancer, Soluble fibrin monomer complex

## Abstract

**Background:**

Esophagectomy is associated with a higher incidence of venous thromboembolism, including pulmonary embolism (PE), than other gastrointestinal cancer surgeries. However, the true incidence of asymptomatic PE remains unclear. This study aimed to determine the incidence and associated factors of PE using routine contrast-enhanced computed tomography (CECT) after minimally invasive esophagectomy (MIE).

**Methods:**

A total of 134 consecutive patients who underwent MIE between May 2022 and December 2023 under a standardized postoperative CECT protocol were retrospectively analyzed. Routine CECT was performed on or after postoperative day (POD) 5 in all patients. Prophylactic anticoagulation was initiated based on bleeding risk. Study endpoints included PE incidence, identification of factors associated with PE, and the discriminative performance of postoperative coagulation and fibrinolysis biomarkers.

**Results:**

PE developed in 13 patients (9.7%), and all cases were asymptomatic. Multivariable analysis identified BMI ≥ 22 kg/m² as independently associated with PE (adjusted OR, 4.53; 95% CI, 1.27–21.4; *P* = 0.019). Sensitivity analyses showed consistent associations across adjusted models. Among postoperative biomarkers, D-dimer on POD 5 showed the highest discriminative performance for PE, whereas soluble fibrin monomer complex (SFMC) on POD 1 showed moderate discrimination.

**Conclusion:**

Despite prophylactic anticoagulation, asymptomatic PE occurred in 9.7% of patients after MIE. BMI ≥ 22 kg/m² was independently associated with PE. Postoperative coagulation and fibrinolysis biomarkers, including POD 5 D-dimer and POD 1 SFMC, demonstrated discriminative performance and may provide complementary information in postoperative evaluation of PE.

**Supplementary Information:**

The online version contains supplementary material available at https://doi.org/10.1007/s10388-026-01224-0.

## Introduction

Radical esophagectomy for esophageal cancer involves extensive lymph node dissection and is associated with substantial postoperative morbidity [1]. Minimally invasive esophagectomy (MIE), including robot-assisted approaches, has been increasingly adopted to reduce surgical invasiveness and improve perioperative outcomes [2, 3]. Nevertheless, venous thromboembolism (VTE) remains a major postoperative complication after esophageal cancer surgery.

VTE encompasses deep vein thrombosis (DVT) and pulmonary embolism (PE), the latter representing a potentially life-threatening event. Previous studies have reported a relatively high incidence of VTE following esophagectomy compared with other gastrointestinal cancer surgeries [4–6]. However, most reports have focused on symptomatic events, and the true incidence of postoperative PE—including asymptomatic cases—remains unclear. Although large database analyses in Japan have reported low rates of symptomatic PE after esophagectomy [7, 8], the true incidence of postoperative PE is likely underestimated when asymptomatic cases are included.

Furthermore, several studies have evaluated postoperative PE using routine contrast-enhanced computed tomography (CECT) after esophagectomy; however, many were conducted without standardized prophylactic anticoagulation, included relatively small sample sizes, or did not perform routine PE screening across the entire cohort [9–12]. Accordingly, independent risk factors for PE in patients undergoing routine imaging under active prophylactic anticoagulation have not been clearly established. In addition, the clinical relevance of postoperative coagulation and fibrinolysis biomarkers in identifying patients at risk for PE remains uncertain.

Therefore, this study aimed to determine the incidence of postoperative PE, including asymptomatic cases, using routine CECT after MIE and to identify factors associated with PE, with additional exploratory assessment of postoperative coagulation and fibrinolysis biomarkers.

## Methods

### Study design and patient population

This observational cohort study was conducted at a single institution. Since May 2022, our institution has implemented a standardized postoperative CECT protocol (DVT-PE protocol encompassing the neck, chest, abdomen, pelvis, and lower extremities) on or after postoperative day (POD) 5 to facilitate early detection of major postoperative complications, including anastomotic leakage and VTE, in patients undergoing MIE, while avoiding unnecessary imaging during the immediate postoperative period. Patients who underwent MIE during this period were retrospectively analyzed within this standardized postoperative imaging protocol.

Between May 2022 and December 2023, a total of 165 consecutive patients underwent MIE, including robot-assisted procedures. Among them, 29 were excluded because routine CECT could not be performed due to contrast allergy, renal dysfunction, or scheduling constraints. Two additional patients who received CECT before POD 4 for clinical indications were excluded to maintain a uniform screening window. The final analytic cohort therefore comprised 134 patients.

### Preoperative management and surgical technique

All patients underwent preoperative thrombosis screening with D-dimer measurement. Patients with D-dimer ≥ 1.0 µg/mL were assessed for DVT and PE using CECT with a DVT-PE protocol when feasible, or lower-extremity ultrasonography if CECT was contraindicated. Patients diagnosed with thrombosis were evaluated by cardiologists and treated with direct oral anticoagulants (DOACs) as indicated. Surgery was scheduled after resolution of thrombosis.

All patients underwent minimally invasive esophagectomy (MIE), including robot-assisted procedures, with two- to three-field lymph node dissection. Reconstruction was performed using a gastric conduit as standard, with a colonic conduit reserved for cases in which gastric reconstruction was not feasible. During the abdominal phase, hand-assisted laparoscopic surgery was employed for gastric conduit reconstruction, whereas open laparotomy was performed for colonic reconstruction. The retrosternal route was the standard reconstruction pathway.

Central venous catheter (CVC) placement was performed in all patients at the completion of surgery. A 4-Fr single-lumen peripherally inserted central catheter (PICC) via the upper arm was preferred. When PICC placement was not feasible due to upper-extremity venous abnormalities or insufficient vessel diameter, a 4-Fr single-lumen CVC was inserted via the femoral vein. Internal jugular vein catheterization was avoided because esophagectomy involves cervical manipulation.

### Postoperative management

VTE prophylaxis followed the Japanese Guidelines for Prevention of Pulmonary Thromboembolism and DVT [13]. Elastic stockings and intermittent pneumatic compression of the lower extremities were applied intraoperatively in all patients. Prophylactic anticoagulation was initiated based on bleeding risk. Low-risk patients received subcutaneous low-molecular-weight heparin (enoxaparin sodium), continuous intravenous unfractionated heparin, or oral DOACs (for prior DOAC users) as early as POD 1. Prophylactic anticoagulation was generally continued for approximately 1 week postoperatively and at least until CVC removal, in accordance with institutional perioperative management practices.

Postoperative care was standardized to minimize venous stasis and hemoconcentration, including same-day extubation and early ambulation from POD 1. Oral intake was initiated on POD 5. Extracellular fluid was administered on POD 0, followed by high-calorie intravenous nutrition via CVC from POD 1. Daily fluid rates were standardized at 1.8 mL/kg/h on PODs 0–1 and 1.6 mL/kg/h from POD 2 onward. CVCs were removed promptly after stabilization of oral intake. Enteral nutrition via nasogastric tube or jejunostomy was not used.

### Contrast-enhanced CT examination and PE/DVT diagnosis

Routine CECT was performed on or after POD 5 according to the institutional protocol. PE was diagnosed when contrast filling defects were observed in the main pulmonary artery or the lobar, segmental, or subsegmental arteries. DVT was diagnosed when thrombi were detected from the inferior vena cava to the lower-extremity veins. All CECT images were independently reviewed by two board-certified radiologists. Asymptomatic PE was defined as PE detected incidentally on routine CECT in the absence of clinical symptoms.

### Endpoints

The primary endpoint was the incidence of PE diagnosed by routine CECT performed on or after POD 5. Secondary endpoints included (i) the incidence of DVT; (ii) identification of factors associated with PE; and (iii) exploratory assessment of postoperative coagulation and fibrinolysis biomarkers—D-dimer, soluble fibrin monomer complex (SFMC), and C-reactive protein (CRP)—in relation to PE.

### Data collection

Patient-related variables included previously reported VTE risk factors in esophagectomy: age, sex, body mass index (BMI), comorbidities (cardiac disease and diabetes mellitus), American Society of Anesthesiologists Physical Status classification, preoperative anticoagulant use, neoadjuvant therapy, clinical stage (cStage; Union for International Cancer Control classification 8th edition), and pathological type [4, 5, 14]. Surgical variables included operative time, intraoperative blood loss, surgical approach, reconstruction organ and route, cervical lymph node dissection, and CVC insertion site [9, 12, 15]. Postoperative variables included anastomotic leakage [≥ Clavien–Dindo (CD) Classification Grade IIIa], pneumonia (≥ CD Grade II), type and timing of prophylactic anticoagulation, CVC removal date, CECT examination date, and length of postoperative hospital stay. Coagulation and fibrinolysis markers (D-dimer, SFMC, and CRP) were measured on PODs 1, 3, 5, and 7.

### Statistical analysis

#### Factors associated with PE

Comparisons between the PE-positive (PE+) and PE-negative (PE−) groups were performed using the Mann–Whitney *U* test for continuous variables and Fisher’s exact test for categorical variables.

To identify preoperative and intraoperative factors associated with PE development, multivariable logistic regression analysis was conducted. Candidate predictors for multivariable analysis were restricted to preoperative and intraoperative variables to avoid adjustment for postoperative factors that may lie on the causal pathway between surgery and PE development. Given the limited number of PE events (*n* = 13), covariate selection was prespecified and restricted to clinically relevant variables to reduce model instability and overfitting. The number of covariates was limited to two per model to maintain an events-per-variable (EPV) ratio ≥ 6.5. Although this is below the conventional EPV threshold of 10, simulation studies have demonstrated acceptable bias and confidence interval (CI) coverage with EPV values between 5 and 9 when effect sizes are not extreme [16, 17]. Accordingly, deliberately parsimonious models were employed.

Age and BMI were selected a priori based on prior associations with postoperative VTE and clinical availability [5, 14]. The BMI cutoff was set at 22 kg/m², corresponding to the “ideal body weight” concept defined by the Japan Society for the Study of Obesity [18]. In addition to the prespecified categorical analysis (BMI ≥ 22 kg/m²), BMI was also analyzed as a continuous variable in a separate multivariable model to assess dose–response relationships and to reduce potential information loss due to dichotomization.

To assess robustness of the association between BMI and PE, sensitivity analyses were performed using separate two-variable models adjusting for established or plausible confounders: (i) operative time (per hour) [15]; and (ii) CVC insertion site (femoral vs. PICC) [12]. Propensity score methods were not applied due to the limited number of PE events (*n* = 13), as reliable estimation of the propensity score and subsequent matching or weighting would be prone to instability and variance inflation in this small-event setting. As a sensitivity analysis, *E*-values were calculated to evaluate the robustness of the observed associations to potential unmeasured confounding [19]. As an additional sensitivity analysis to mitigate small-sample and rare-event bias, Firth penalized logistic regression was performed. Results are presented as odds ratios (ORs) with 95% CIs.

### Biomarker analysis

Receiver operating characteristic (ROC) analyses for biomarker discrimination were performed in R using the pROC package. Biomarker analyses were exploratory and performed in a hypothesis-generating manner. ROC analyses were conducted for all prespecified biomarkers, including D-dimer, SFMC, and CRP, at each postoperative time point (PODs 1, 3, 5, and 7). Area under the curve (AUC) CIs were estimated, and statistical significance versus an AUC of 0.5 was assessed using DeLong’s test. Optimal cutoffs were determined using the Youden index within the study cohort, and sensitivity, specificity, positive predictive value, and negative predictive value were calculated using these derived thresholds. Given the limited number of events, *P *values from ROC analyses were interpreted descriptively without formal adjustment for multiple comparisons.

All statistical tests were two-sided, and *P *value < 0.05 was considered statistically significant. Primary analyses were conducted using JMP Student Edition 18 (SAS Institute Inc., Cary, NC, USA), while ROC analyses and Firth penalized logistic regression were performed in R (version 4.3.1; R Foundation for Statistical Computing, Vienna, Austria).

## Results

### PE/DVT incidence and patient characteristics

Among the 134 patients analyzed, PE developed in 13 (9.7%). All PE events were distal to the lobar arteries and asymptomatic, having been incidentally identified via routine CECT screening. No fatal PE occurred in any patient, and all affected patients were managed on general wards without intensive care. DVT developed in 10 patients (7.5%), including concurrent PE and DVT in 2 patients (1.5%).

Preoperative VTE was identified in seven patients, all of whom received anticoagulation therapy before surgery. The median duration of preoperative anticoagulation therapy was 37 days (range, 17–67). Surgery was scheduled after radiological confirmation of thrombus resolution, and no patients experienced clinically significant oncological disadvantage due to thrombosis treatment.

The PE + and PE− groups had median ages of 64 and 68 years and male proportions of 11 (85%) and 99 (82%), respectively. Median BMI was significantly higher in the PE+ group than in the PE− group (23.5 vs. 21.5 kg/m², *P* = 0.044). Femoral CVC insertion was more common in the PE+ group (7 patients, 54%) than in the PE− group (39 patients, 32%), although the difference did not reach statistical significance (*P* = 0.13). Initiation of prophylactic anticoagulation tended to be later in the PE+ group (*P* = 0.14), whereas CVC removal tended to occur earlier (*P* = 0.060), although neither difference reached statistical significance (Table 1).

**Table 1:** PE/DVT incidence, patient characteristics, and perioperative factors

Variable	Total (*n* = 134)	PE + group (*n* = 13)	PE - group (*n* = 121)	*P* value
**PE/DVT incidence**				

DVT	10 (7.5)	2 (15)	8 (6.6)	0.25
Both PE and DVT	2 (1.5)	2 (15)	0 (0)	0.009

**Patient characteristics**				

Age	68 [59–75]	64 [56–74]	68 [59–75]	0.27
Sex				1.0
Male	110 (82)	11 (85)	99 (82)	
Female	24 (18)	2 (15)	22 (18)	
BMI	21.7 [19.4–23.7]	23.5 [20.5–26.6]	21.5 [19.3–23.5]	0.044
Cardiac disease	56 (42)	4 (31)	52 (43)	0.56
Diabetes mellitus	19 (14)	1 (8)	18 (15)	1.0
ASA-PS				1.0
1–2	116 (87)	12 (92)	104 (86)	
3	3 (13)	1 (8)	17 (14)	
Preoperative anticoagulant use	27 (20)	4 (31)	23 (19)	0.47
Neoadjuvant theraphy				0.67
NAC	101 (75)	9 (69)	92 (76)	
CRT	9 (7)	1 (8)	8 (7)	
None	24 (18)	3 (23)	21 (17)	
cStage				0.15
I, II	56 (42)	8 (62)	48 (40)	
III, IV	78 (58)	5 (38)	73 (60)	
Pathological type				0.13
SCC	124 (93)	11 (85)	113 (93)	
Adenocarcinoma	8 (6)	1 (8)	7 (6)	
Others	2 (1)	1 (8)	1 (1)	

**Operative factors**				

Operative time, min	491 [410–585]	507 [437–646]	490 [400–582]	0.27
Blood loss, mL	170 [100–285]	172 [138–231]	170 [94–287]	0.71
Surgical approach				1.0
MIE	97 (72)	10 (78)	87 (72)	
RAMIE	37 (28)	3 (23)	34 (28)	
Reconstruction organ				1.0
Stomach	130 (97)	13 (100)	117 (97)	
Colon	4 (3)	0	4 (3)	
Reconstruction route				0.52
Retrosternal	127 (95)	12 (92)	115 (95)	
Posterior mediastinal	7 (5)	1 (8)	6 (5)	
Cervical lymph node dissection	44 (33)	4 (33)	40 (33)	1.0
CVC insertion site				0.13
PICC (upper arm)	88 (66)	6 (46)	82 (68)	
CVC (femoral)	46 (34)	7 (54)	39 (32)	

**Post operative factors**				

Post operative complications				
Anastomotic leakage	2 (2)	1 (8)	1 (1)	0.19
Pneumonia	7 (5)	0	7 (6)	1.0
Prophylactic anticoagulation use	106 (79)	10 (77)	96 (79)	0.73
Type of prophylactic anticoagulation				0.73
Enoxaparin sodium	83 (62)	6 (46)	77 (64)	
Heparin sodium	19 (14)	3 (23)	16 (13)	
DOAC	4 (3)	1 (8)	3 (2)	
None	28 (21)	3 (23)	25 (21)	
Prophylactic anticoagulation start (POD)	2 [2–3]	3 [2–4]	2 [2–3]	0.14
CVC removal (POD)	6 [6–7]	6 [5–6]	6 [6–7]	0.06
Contrast-enhanced CT (POD)	7 [6–7]	7 [6–7]	7 [7–7]	0.98
Postoperative hospital stay, days	16 [12–23]	16 [13–18]	15 [12–24]	0.68

*PE* pulmonary embolism, *DVT* deep vein thrombosis, *IQR* interquartile range, *BMI* body mass index, *ASA-PS* American Society of Anesthesiologists Physical Status, *NAC* neoadjuvant chemotherapy, *CRT* chemoradiotherapy, *SCC* squamous cell carcinoma, *MIE* minimally invasive esophagectomy, RAMIE robot-assisted minimally invasive esophagectomy, *CVC* central venous catheter, *PICC* peripherally inserted central catheter, *DOAC* direct oral anticoagulant, and *POD* postoperative day.

Continuous variables are presented as median [IQR], and categorical variables as *n* (%).

### Multivariable analysis of factors associated with PE

In the primary multivariable model, BMI ≥ 22 kg/m² was independently associated with PE (adjusted OR, 4.53; 95% CI, 1.27–21.4; *P* = 0.019; *E*-value, 8.5), whereas age was not associated. When BMI was modeled as a continuous variable adjusted for age, it remained significantly associated with PE (adjusted OR per 1 kg/m² increase, 1.27; 95% CI, 1.04–1.59; *P* = 0.018), supporting a dose–response relationship. In sensitivity analysis Model 1, adjusting for operative time (per hour), BMI remained significant (adjusted OR, 4.09; 95% CI, 1.14–19.3; *P* = 0.030; *E*-value, 7.7), whereas operative time was not associated. In sensitivity analysis Model 2, adjusting for CVC site, BMI remained significant (adjusted OR, 4.63; 95% CI, 1.27–22.1; *P* = 0.019; *E*-value, 8.7), and femoral CVC insertion was independently associated with PE (adjusted OR, 3.58; 95% CI, 1.04–13.3; *P* = 0.044). *E*-values consistently exceeded 7.7, indicating that substantial unmeasured confounding would be required to fully explain the observed association (Table 2). Firth penalized logistic regression yielded similar effect estimates and did not materially alter the conclusions (Online Resource 1).

**Table 2:** Multivariable logistic regression analysis of factors associated with PE, including sensitivity analyses

Model	Variable	Adjusted OR	95% CI	*P* value
**Primary model**	BMI ≥ 22 kg/m² (vs. < 22)	4.53	1.27–21.4	0.019
(categorical BMI)	Age (per year)	0.97	0.91–1.03	0.27

**Alternative specification**	BMI (per 1 kg/m² increase)	1.27	1.04–1.59	0.018
(continuous BMI)	Age (per year)	0.97	0.91–1.03	0.34

**Sensitivity analysis Model 1**	BMI ≥ 22 kg/m² (vs. < 22)	4.09	1.14–19.3	0.030
	Operative time (per hour)	1.21	0.89–1.69	0.24

**Sensitivity analysis Model 2**	BMI ≥ 22 kg/m² (vs. < 22)	4.63	1.27–22.1	0.019
	Femoral CVC (vs. PICC)	3.58	1.04–13.3	0.044

*PE* pulmonary embolism, *BMI* body mass index, *CVC* central venous catheter, *PICC* peripherally inserted central catheter, *OR* odds ratio, and *CI* confidence interval.

### Discriminative performance of coagulation and fibrinolysis biomarkers for PE

SFMC and CRP levels on POD 1, as well as D-dimer levels on PODs 3, 5, and 7, were significantly higher in the PE+ group than in the PE− group. ROC curve analysis using DeLong’s test showed that D-dimer on POD 5 had the highest discriminative performance (AUC, 0.76; 95% CI, 0.61–0.90; *P* < 0.001). At the optimal cutoff (6.1 µg/mL), sensitivity and specificity were 76.9% and 76.6%, with positive and negative predictive values of 26.3% and 96.8%, respectively. D-dimer on POD 7 also demonstrated moderate discriminative performance (AUC, 0.74; 95% CI, 0.59–0.88; *P* = 0.001). SFMC on POD 1 demonstrated moderate discriminative performance (AUC, 0.71; 95% CI, 0.55–0.86; *P* = 0.008). At the optimal cutoff (20.1 µg/mL), sensitivity, specificity, positive predictive value, and negative predictive value were 76.9%, 58.8%, 16.9%, and 95.9%, respectively (Table 3).

**Table 3:** Discriminative performance of postoperative coagulation and fibrinolysis biomarkers for PE

Variable	PE+ group (*n* = 13)	PE - group (*n* = 121)	*P* value
**D-dimer**			
POD1	6.3 [5.0–12.4]	4.9 [3.9–8.3]	0.082
POD3	5.4 [3.6–6.6]	3.6 [3.0–5.1]	0.032
POD5	7.1 [5.6–8.5]	4.6 [3.5–5.9]	0.002
POD7	7.8 [5.8–10.2]	5.2 [3.9–6.9]	0.005
**SFMC**			
POD1	41 [17.1–98.2]	17.7 [11.8–30.1]	0.041
POD3	28 [18.4–100]	26.4 [17.9–47.1]	0.53
POD5	21.1 [12.7–86.4]	16.4 [11.2–29.0]	0.17
POD7	9.9 [8.3–17.0]	11 [8.3–18.1]	0.90
**CRP**			
POD1	6 [4.5–7.1]	4.6 [3.7–5.8]	0.041
POD3	11.2 [6.6–16.0]	8.7 [6.0–13.4]	0.33
POD5	4.5 [2.8–10.3]	4.6 [2.3–6.7]	0.35
POD7	4.1 [2.1–6.6]	2.6 [1.4–5.3]	0.14

	AUC	*P* value	95% CI	Cutoff value	Sensitivity (%)	Specificity (%)	PPV (%)	NPV (%)
D-dimer POD 3	0.68	0.047	0.50–0.86	4.3 (μg/mL)	76.9	70	21.7	96.6
D-dimer POD 5	0.76	<0.001	0.61–0.90	6.1 (μg/mL)	76.9	76.6	26.3	96.8
D-dimer POD 7	0.74	0.001	0.59–0.88	6.4 (μg/mL)	76.9	68.3	20.8	96.5
SFMC POD 1	0.71	0.008	0.55–0.86	20.1 (μg/mL)	76.9	58.8	16.9	95.9
CRP POD 1	0.67	0.020	0.53–0.82	5.9 (mg/dL)	61.5	75.8	21.6	94.8

*IQR* interquartile range, *PE* pulmonary embolism, *ROC* receiver operating characteristic, *SFMC* soluble fibrin monomer complex,*POD* postoperative day, *AUC* area under the curve, *PPV* positive predictive value, and *NPV* negative predictive value.

Upper panel shows between-group comparisons of biomarkers. Lower panel shows ROC analysis results.

Continuous variables are presented as median [IQR].

## Discussion

In this observational cohort study incorporating routine CECT after MIE, postoperative PE was detected in 9.7% of patients despite prophylactic anticoagulation, and all cases were asymptomatic. Previous Japanese database studies reported relatively low rates of symptomatic PE after esophagectomy [7, 8], whereas studies using routine postoperative imaging reported higher incidences of asymptomatic events [9–11]. These findings suggest that asymptomatic PE may occur even under active prophylactic anticoagulation, indicating that residual thromboembolic risk may persist despite contemporary prophylactic strategies.

Several studies have assessed asymptomatic PE after esophageal cancer surgery using routine imaging. Kato et al. [9] and Nakamura et al. [10] reported PE incidences of 3.9% and 24.6%, respectively, in cohorts without standardized prophylactic anticoagulation, whereas Yoshida et al. [11] observed a lower incidence in a small cohort receiving enoxaparin sodium. Torii et al. [12] examined venous thrombosis risk factors but did not specifically evaluate PE using routine screening. These reports were limited by either lack of standardized prophylaxis or small sample size and did not consistently evaluate independent PE risk factors. In contrast, this study analyzed PE incidence and associated factors in a relatively large cohort undergoing routine imaging under active prophylactic anticoagulation, providing insight into residual thromboembolic risk in contemporary perioperative practice.

The clinical significance of asymptomatic PE, particularly small subsegmental PE, remains debated [20]. However, incidental PE in patients with cancer is frequently managed with anticoagulation in clinical practice, and current guidelines recommend short-term anticoagulation rather than observation in most cases [21]. Given the prothrombotic milieu associated with malignancy and surgical stress [22], detection of otherwise unrecognized PE may have implications for postoperative management strategies.

BMI ≥ 22 kg/m² was the only factor that remained significantly associated with PE across prespecified multivariable and sensitivity analyses. Age and operative time were not independently associated with PE. Although advanced age has previously been identified as a risk factor for postoperative VTE [5], and operative time has been linked to symptomatic thromboembolic events [15], obesity is a well-established risk factor for VTE in general populations [23]. In this context, our findings suggest that metabolic factors may play a more prominent role in the development of asymptomatic PE after esophagectomy. Notably, the association persisted when BMI was analyzed as a continuous variable, supporting a dose–response relationship rather than a threshold-dependent effect. The identified BMI cutoff may be particularly relevant in Asian populations, in whom metabolic abnormalities tend to occur at lower BMI levels [24]. Adipose tissue–related procoagulant activity provides biological plausibility for this association [25]. From a clinical perspective, BMI ≥ 22 kg/m² is readily determined preoperatively and may aid risk stratification. Patients with BMI ≥ 22 kg/m² may benefit from earlier initiation of pharmacologic prophylaxis when clinically feasible, as well as closer postoperative surveillance.

In exploratory analyses, femoral CVC insertion was more frequent in the PE+ group after adjustment for BMI, although this was not observed in univariate analysis. Prior studies have described differing complication profiles across catheter insertion sites without consistent differences in PE incidence [26, 27]. This finding may reflect confounding related to patient characteristics influencing catheter selection. Although residual confounding cannot be excluded, avoidance of femoral venous catheterization when clinically feasible may be considered as a potential strategy to mitigate thromboembolic risk.

Among postoperative biomarkers, D-dimer on POD 5 demonstrated the highest discriminative performance with a high negative predictive value. Because routine CECT was performed at a median of POD 7, POD 5 D-dimer values were generally available prior to imaging, suggesting potential utility for pre-imaging triage. D-dimer is widely accessible in clinical practice; however, its specificity in the acute postoperative setting may be reduced, as levels can be elevated by inflammation, tissue injury, and perioperative stress [28]. SFMC on POD 1 showed moderate discriminative ability with a similarly high negative predictive value. As SFMC reflects early fibrin formation preceding D-dimer elevation [29], POD 1 values may provide early information on postoperative coagulation activation. However, given the low positive predictive values observed, elevation of D-dimer or SFMC should not, in isolation, prompt therapeutic anticoagulation. Nevertheless, biomarker measurements may provide complementary information when integrated with clinical assessment.

The proportion of patients receiving prophylactic anticoagulation did not differ between the PE + and PE− groups, whereas initiation tended to be delayed in the PE+ group. Intensified anticoagulation may reduce thrombotic events; however, this must be balanced against bleeding risk after esophagectomy. These findings suggest that baseline clinical factors, such as BMI, may help inform risk stratification when planning prophylactic strategies rather than relying solely on uniform approaches.

This study has several limitations. First, it was conducted at a single center with only 13 PE events, which may limit statistical precision despite the use of sensitivity analyses and Firth penalized regression. Second, imaging was performed on or after POD 5 (median POD 7). Because only a single CECT examination was performed as part of the institutional postoperative protocol, PE events occurring later in the postoperative course may not have been detected. Therefore, a negative finding on this single postoperative CECT does not completely exclude the possibility of subsequent thrombus formation. Third, ROC-derived cutoff values were internally generated and therefore require external validation. Larger multicenter prospective studies are required to validate these findings and to clarify the clinical implications of postoperative biomarker assessment.

## Conclusion

Routine CECT identified asymptomatic PE in 9.7% of patients after MIE despite prophylactic anticoagulation. BMI ≥ 22 kg/m² was independently associated with PE. Postoperative coagulation and fibrinolysis biomarkers, including POD 5 D-dimer and POD 1 SFMC, demonstrated discriminative performance and may provide complementary information in postoperative evaluation of PE.

## Supplementary Information

Below is the link to the electronic supplementary material.

Supplementary Material 1

## Data Availability

The datasets generated and/or analyzed during the current study are not publicly available due to privacy and ethical restrictions, but are available from the corresponding author on reasonable request.
