# Supplementary material for: Routine contrast-enhanced CT screening identifies asymptomatic pulmonary embolism after minimally invasive esophagectomy: incidence and associated factors
Source: Esophagus. 2026 Jun 30;23(4):896–904. doi: 10.1007/s10388-026-01224-0 (PMC13723685; doi:10.1007/s10388-026-01224-0)
Supplement: Supplementary file 1 [file 10388_2026_1224_MOESM1_ESM.pdf]

Article title: Routine contrast-enhanced CT screening identifies asymptomatic pulmonary embolism after minimally invasive esophagectomy: incidence and associated factors

Journal: Esophagus

Authors: Akira Saito, et al.

Corresponding author:
Akira Saito, M.D., Ph.D.
Division of General and Gastroenterological Surgery, Department of Surgery, Showa Medical University School of Medicine, 1-5-8, Hatanodai, Shinagawa, Tokyo, 142-8666, JAPAN
Email: asaito1205@med.showa-u.ac.jp

Online Resource 1. Firth penalized logistic regression analysis of PE

Model	Variable	Adjusted OR	95% CI	*P* value
**Primary model**	BMI ≥ 22 kg/m² (vs. < 22)	3.99	1.19–16.7	0.024
(categorical BMI)	Age (per year)	0.97	0.91–1.03	0.29
**Alternative specification**	BMI (per 1 kg/m² increase)	1.26	1.04–1.55	0.018
(continuous BMI)	Age (per year)	0.97	0.91–1.03	0.29
**Sensitivity analysis Model 1**	BMI ≥ 22 kg/m² (vs. < 22)	3.63	1.08–15.2	0.037
	Operative time (per hour)	1.20	0.88–1.66	0.24
**Sensitivity analysis Model 2**	BMI ≥ 22 kg/m² (vs. < 22)	4.10	1.21–17.4	0.023
	Femoral CVC (vs. PICC)	3.40	1.04–11.9	0.043

Conducted as a sensitivity analysis to address small-sample bias.

PE pulmonary embolism, BMI body mass index, CVC central venous catheter,

PICC peripherally inserted central catheter, OR odds ratio, and CI confidence interval.
